# Multifaceted roles of microRNAs: From motor neuron generation in embryos to degeneration in spinal muscular atrophy

**DOI:** 10.7554/eLife.50848

**Published:** 2019-11-18

**Authors:** Tai-Heng Chen, Jun-An Chen

**Affiliations:** 1 PhD Program in Translational Medicine, Graduate Institute of Clinical MedicineKaohsiung Medical University, Academia SinicaKaohsiungTaiwan; 2 Department of Pediatrics, Division of Pediatric EmergencyKaohsiung Medical University Hospital, Kaohsiung Medical UniversityKaohsiungTaiwan; 3 Institute of Molecular BiologyAcademia SinicaTaipeiTaiwan; 4 Faculty of Medicine, College of MedicineKaohsiung Medical UniversityKaohsiungTaiwan; California Institute of TechnologyUnited States

**Keywords:** Developmental Biology, Human Biology and Medicine, Neuroscience, Stem Cell and Regenerative Medicine

## Abstract

Two crucial questions in neuroscience are how neurons establish individual identity in the developing nervous system and why only specific neuron subtypes are vulnerable to neurodegenerative diseases. In the central nervous system, spinal motor neurons serve as one of the best-characterized cell types for addressing these two questions. In this review, we dissect these questions by evaluating the emerging role of regulatory microRNAs in motor neuron generation in developing embryos and their potential contributions to neurodegenerative diseases such as spinal muscular atrophy (SMA). Given recent promising results from novel microRNA-based medicines, we discuss the potential applications of microRNAs for clinical assessments of SMA disease progression and treatment.

## Introduction

How neurons establish individual identity in the developing nervous system and why only specific neuron subtypes are susceptible to degeneration in neurodegenerative disease are two challenging unanswered questions in neuroscience. Answers to these questions are crucial to establishing the patterns of connectivity between neuronal types and their selective targets. Deciphering neuronal subtype is relatively straightforward in *Caenorhabditis elegans*, as each neuronal lineage and its position, pattern of connectivity, molecular profile, and function have all been well characterized (Hobert, 2008). However, the complex array of neurons in the vertebrate nervous system (e.g., 10^11^ in the human brain) renders it rather more challenging to define a neuronal type. Thus, a clear neuronal taxonomy that completely reconciles morphological, physiological, molecular, and perhaps other criteria (e.g., position, connectivity) is essential. Neuronal diversity in the central nervous system (CNS) was vividly described by Sir Roger Penrose: “*If you look at the entire physical cosmos, our brains are a tiny, tiny part of it. But they're the most perfectly organized part. Compared to the complexity of a brain, a galaxy is just an inert lump*’.

**Figure 1.**
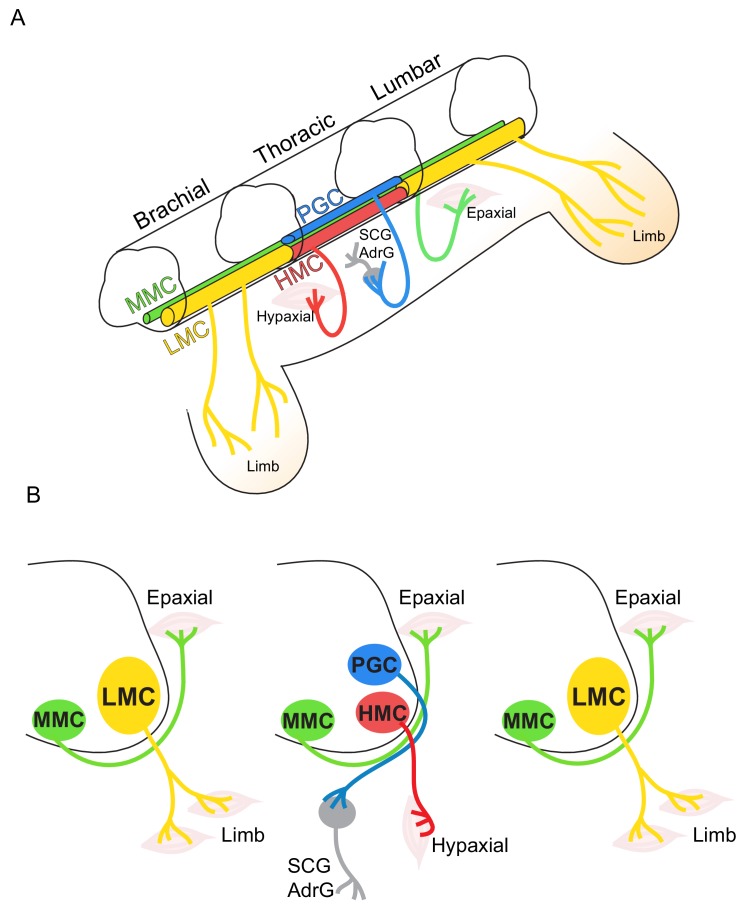
Topographic distribution of motor neuron (MN) columnar identities along the rostrocaudal axis of the spinal cord. (**A**) Schematic illustration of the three-dimensional arrangement of motor columns in the spinal cord. Limb-innervating MNs are located in the lateral motor columns (LMC) at brachial and lumbar levels, whereas preganglionic (PGC) and hypaxial (HMC) neurons at thoracic levels innervate the sympathetic ganglia and hypaxial muscles, respectively. Medial motor (MMC) neurons are distributed at all rostrocaudal levels and project dorsally to epaxial muscles. (**B**) Diagram illustrating transverse sections through each segment, depicting the directions of MN axonal projection to the muscle targets.

The mechanisms leading to neuronal diversity in the CNS is arguably best characterized by spinal motor neurons (MNs). Within the vertebrate spinal cord, the cell bodies of MNs innervating specific muscle targets are topographically organized within columnar, divisional, and pool subtypes (Lance-Jones and Landmesser, 1981; Landmesser, 1978a; Landmesser, 1978b). Spinal MNs are diversified into hundreds of subtypes along the rostrocaudal (RC) axis (Figure 1) (Catela et al., 2015). Spinal MNs are located in the ventral horn of the spinal cord and transmit signals from the brain to innervated muscle targets in the periphery to control all muscle movements. The human body contains more than 300 bilateral pairs of muscles and ~100 million muscle fibers. The control of muscular contractions for different motor behaviors relies on MN diversity (Catela et al., 2015; Kanning et al., 2010). Stereotypic axonal projection by MNs suggests the existence of MN subtype identities, which would facilitate selectivity in innervation patterns of different muscle targets. MNs can find their original targets even if they have been displaced to another segment of the spinal cord before axonal extension, which indicates that most MNs are programmed with an intrinsic identity that encodes information on their targeting preference (Lance-Jones and Landmesser, 1980). Moreover, MN subtypes exhibit differential vulnerability in MN-related degenerative diseases that cause progressive muscle weakness and paralysis, such as adult-onset amyotrophic lateral sclerosis (ALS) and childhood spinal muscular atrophy (SMA), further support an intrinsic susceptibility to degeneration amongst MN subtypes. Thus, a comprehensive understanding of the underlying mechanisms for MN diversification will likely shed light on MN target selectivity, subtype survival and differential vulnerability to MN-related diseases.

**Figure 2.**
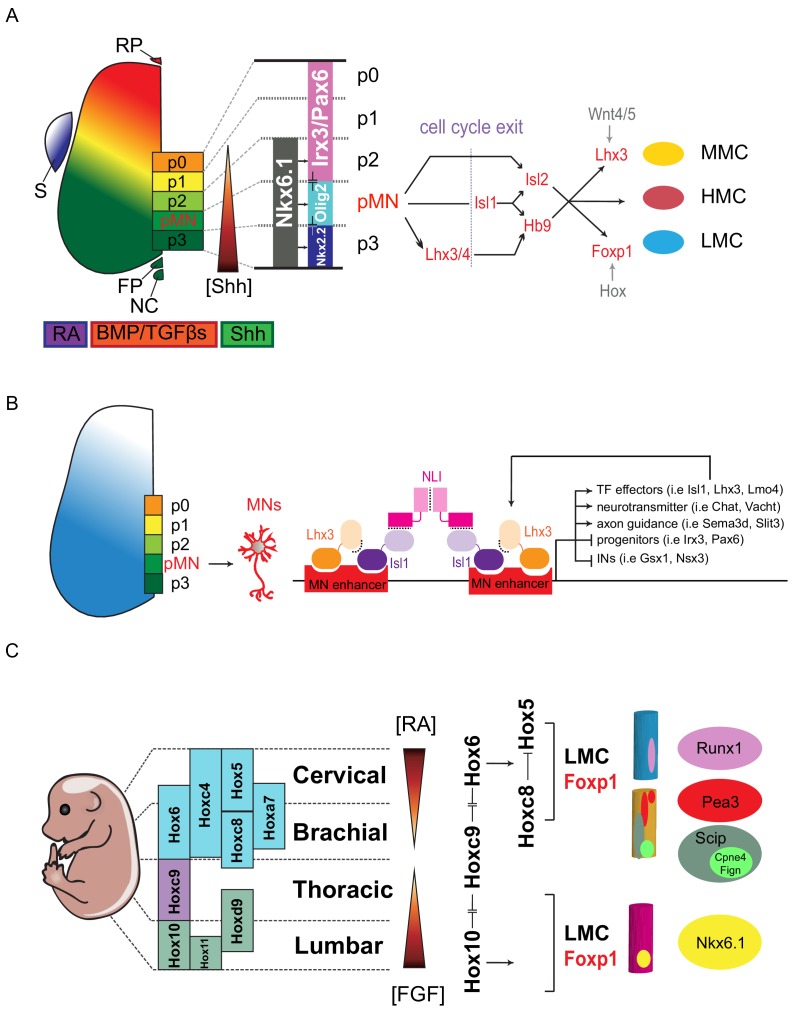
Transcription factor-based dorsoventral (DV) and rostrocaudal (RC) pattering of the spinal cord. (**A**) Upon neural tube closure, a gradient of Sonic hedgehog protein (Shh) emanating from the notochord (NC) and floor plate (FP), together with BMP/TGFβ signaling from the roof plate (RP), mediates repression of class I homeodomain proteins (e.g. Irx3 and Pax6) and induction of class II protein expression (e.g. Nkx6.1 and Nkx2.2) at different threshold concentrations. Retinoic acid (RA) is expressed by the paraxial mesoderm to induce expression of class I proteins, which are turned off more ventrally by threshold levels of Shh. Class I and class II proteins that abut each other to define progenitor domain boundary respectively. Shh signaling defines five progenitors (p0-p3 and pMN) that give rise to V0-V3 interneurons and motor neurons. S, somite; RP, roof plate; BMP, bone morphogenetic proteins; TFGβs, transforming growth factor beta. (**B**) Isl1–Lhx3 forms MN-hexamer complexes to direct the differentiation of MNs by binding to MN enhancers. Isl1–Lhx3 induces the expression of a battery of MN genes that give rise to functional hallmarks of MNs, while suppressing key interneuron genes. Furthermore, the Isl1-Lhx3 complex amplifies its own expression through a potent autoregulatory feedback loop and simultaneously enhances the transcription of Lmo4 to promote MN differentiation and maturation. (**C**) Fibroblast growth factor (FGF) signals maintain a caudal progenitor zone during axis extension, and down-regulation of FGF signaling by RA is required for neuronal patterning and differentiation at more rostral levels. The RA and FGF gradient elicits expression of Hox genes along the rostrocaudal axis. Similar to patterning along the dorsoventral axis, the coordinated interactions between Hox family members allow regional boundaries to be delineated. Specific expression of Hox accessory factors, such as Foxp1, can further specify MN columnar subtypes. Within lateral motor columns, MN pools that innervate different limb muscle types can be defined by hallmark transcription factors (TFs) such as Runx1, Pea3, Scip, Nkx6.1, as well as digit-innervating MNs that manifest Fign, Cpne4, or other TFs.

Spinal MNs are delineated according to the combinatorial actions of three homeodomain-containing transcription factors (TFs) – Nkx2.2, Nkx6.1 and Irx3 – that confine the generation of motor neuron progenitors (pMNs) to the appropriate region of the neural tube (Balaskas et al., 2012; Briscoe et al., 2000) (Figure 2A). Nkx2.2 and Irx3 initially ensure that pMNs are induced in the ventral spinal cord. Once pMNs have been defined, Nkx6.1 activity promotes the pMN-restricted induction of downstream factors, such as the basic helix-loop-helix (bHLH) factor Olig2 (Novitch et al., 2001). Olig2 has a dual role in coordinating the acquisition of pan-neuronal properties and subtype characteristics of differentiating MNs. Forced expression of Olig2 in the dorsal region is sufficient to repress Irx3. Recent studies have further revealed the gene-regulatory network (GRN) of these progenitor TFs responsible for establishing cell identities in the developing spinal cord, with progenitor domains being determined by a network of transcriptional repressors that repress all inappropriate and alternative cell fates to elicit a single definitive identity (Figure 2A) (Kutejova et al., 2016). Additionally, progenitor domain-specific master TFs also directly repress ‘effector’ genes expressed in other progenitor domains. For example, pMN-master regulator Olig2 directly represses alternative interneuron domain TFs such as Pax6, Irx3, and Nkx2.2. In *Olig2* mutants, Pax6 and Nkx2.2 are ectopically expanded into pMNs, supporting the concept of cross-repressive TF-mediated GRN in the pMNs (Balaskas et al., 2012; Zhou and Anderson, 2002).

After pMNs have been defined within the neural tube, both cell proliferation and cell cycle exit are patterned in time and space to regulate MN subtypes (Kicheva et al., 2014). In anamniotes, maintenance of pMNs also requires an MN-specific Cyclin Dx (Ccndx). Inhibition of Ccndx results in specific loss of differentiated MNs (Chen et al., 2007; Lien et al., 2016). Given that overexpression of D-type cyclin in amniotes appears insufficient to alter the cell-fate decision and timing of neuronal differentiation in the spinal cord, it is not clear if higher vertebrates need specific cyclins to replenish pMNs to generate the diverse subtypes of MNs and oligodendrocytes (Lobjois et al., 2008). In mouse embryos, spinal MNs acquire generic MN identity after cell cycle exit (i.e., at about embryonic day 9.5), and express a common set of TFs – Mnx1 (Hb9), Lhx3, Isl1 and Isl2 (Novitch et al., 2001; Thaler et al., 1999; Tsuchida et al., 1994) (Figure 2A). These MNs project axons outside the spinal cord to peripherals and release acetylcholine as a neurotransmitter. Generic spinal MN identity is established by cooperative binding of the LIM complex comprising Isl1 and Lhx3 to MN-specific enhancers, thereby inducing the expression of a battery of MN genes that induce functional hallmarks of MNs, while suppressing key interneuron genes. Furthermore, the Isl1-Lhx3 complex amplifies its own expression through a potent autoregulatory feedback loop and simultaneously enhances the transcription of *Lmo4* to promote MN differentiation and maturation (Erb et al., 2017; Lee et al., 2012) (Figure 2B).

As embryonic development progresses, MNs diversify to exhibit subtype identities. Establishment of MN subtype is mediated by mutually exclusive expression of Hox TFs, which is programmed according to body segment along the RC axis. For example, segmental identity of MNs is defined by the mutually exclusive expression of Hox6, Hox9 and Hox10 (Dasen et al., 2003; Lacombe et al., 2013) (Figure 2C). In particular, a single *Hox* gene, *Hoxc9*, required for the generation of thoracic MN subtypes, is essential for organizing the MN topographic map, and acts as a key repressor of the forelimb-level *Hox* network (Jung et al., 2010). In each segment, MNs are grouped into different columns according to their innervating targets. For instance, within the brachial segment hosting Hox6 expression, MNs are further grouped into axial muscle-projecting MNs (Lhx3^on^, MMC) and forelimb-innervating MNs (Foxp1^on^, LMC)(Dasen et al., 2008). Within Hoxc6^on^ MNs, expression of another set of mutually exclusive Hox proteins, such as Hox5 and Hox8 in Foxp1^on^ LMC, controls the rostral and caudal motor pool identity, which directs motor pools to either innervate proximal or distal muscles in the forelimb (Catela et al., 2015). Although a mutually exclusive expressed Hoxa5-Hoxc8 sharp boundary is manifested within the Hoxc6^on^ MNs, unlike progenitor-TFs that display mutual inhibitions, only Hoxc8 represses Hoxa5 unilaterally at protein level (Dasen et al., 2005; Li et al., 2017). It is therefore expected that additional Hox-mediated GRNs might be needed to consolidate the robust boundary within the brachial LMC-MNs.

LMC is the most extensively studied motor column, and this motor column is further classified into divisional identities targeting flexor and extensor limb muscles (Kania and Jessell, 2003; Sockanathan and Jessell, 1998). Further heterogeneity in spinal MNs can be revealed by retrograde labeling of each muscle group, with labeled MNs occupying distinct and stereotyped positions in the spinal cord (Hollyday, 1980a; Hollyday, 1980b; Landmesser, 1978a; Landmesser, 1978b; McHanwell and Biscoe, 1981; Romanes, 1964; Sürmeli et al., 2011; Vanderhorst and Holstege, 1997). Therefore, MN subtypes can be further assigned into pool identity according to innervated muscle targets. It is believed that one of the physiological functions of MN pool diversification during development is to enable precise target selection. However, it remains unclear how each MN subtype establishes its selectivity to target distinct muscle fibers. Many studies have revealed that a number of proteins – including LIM-HD, bHLH, Hox and ETS-TFs – play major roles in establishing MN subtype identity and connectivity during development (Dasen et al., 2005; Kania and Jessell, 2003; Tsuchida et al., 1994; Vermot et al., 2005) (Figure 2C). For example, Nkx6.1 and Nkx6.2 are expressed in subpopulations of MNs and loss-of-function studies of these proteins have resulted in target innervation and arborization defects in adductor and gracilis muscles (De Marco Garcia and Jessell, 2008; Ghosh and Kolodkin, 1998; Livet et al., 2002). In addition, extrinsic cues are also indispensable for precisely defining MN subtype diversification and axon targeting. For example, expression of the ETS-TF *Etv4* (Pea3) in MNs innervating cutaneous maximus and latissimus dorsi muscles in the forelimb requires GDNF signal activation from the periphery (Haase et al., 2002). Recently, Jessell and colleagues revealed that *Cpne4*/*Fign* markers and evasion of non-canonical retinoic acid signaling label the MN subtype innervating digit muscles, providing insights into the emergence of a divergent molecular program during evolution (Mendelsohn et al., 2017). Even though 60 motor pools are thought to exist in either the brachial or lumbar LMC-MNs innervating mouse limb muscles, a complete subtype-specific gene profile for each motor pool is still lacking, not to mention for the other less well-profiled motor columns (D’Elia and Dasen, 2018; Fetcho, 1987) (Figure 2B).

Above all, spinal MN diversity is initiated by differential distribution of extrinsic morphogen signals, which elicit a set of cardinal TFs to abut each other and consolidate the cell fate of pMNs along the dorsoventral (DV) axis. Consequently, MN subtypes are distributed topographically along the RC axis in the spinal cord to align innervating muscle targets during development. Although the morphogen-mediated GRN can explain most of the principles leading to MN diversity, it is still not adequate to establish how cells at the boundaries exposed to similar morphogen thresholds adopt an ‘all or none’ choice. Furthermore, TF-mediated cell fate determination is insufficient for generating a finer resolution of MN subtypes within a particular segment, so other regulatory pathways must be employed.

## MicroRNAs during motor neuron development

**Figure 3.**
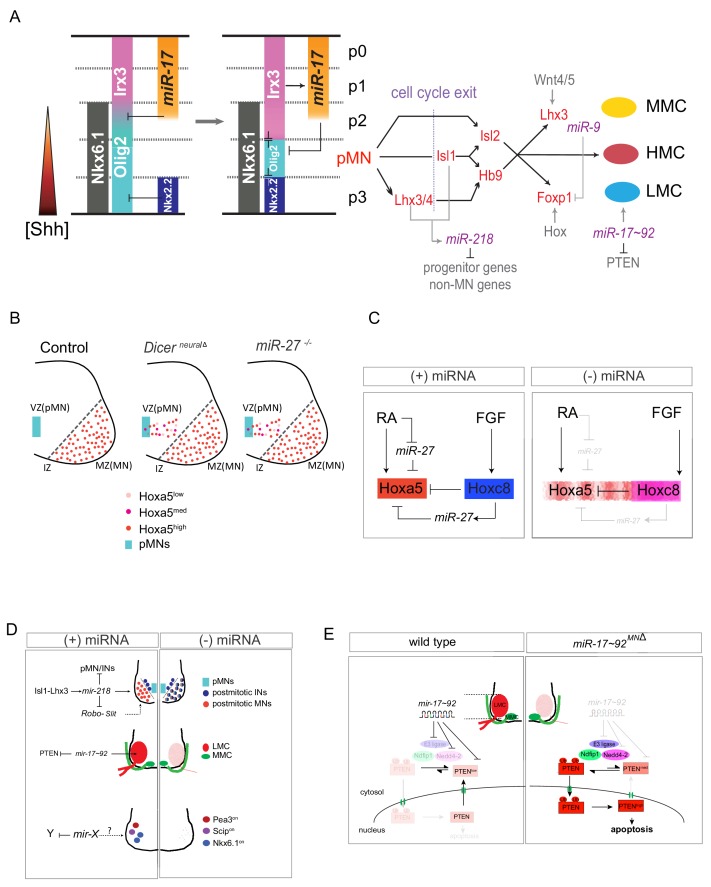
MicroRNA-mediated dorsoventral (DV) and rostrocaudal (RC) pattering of the spinal cord. (**A**) In early embryonic stages (E8.5~E9), Nkx6.1 and Olig2 are co-expressed in a broad ventral domain spanning the prospective p3, pMN and part of the p2 domains. Subsequently, Nkx2.2 induced by sustained Shh signaling represses Olig2 in the p3 domain and *miR-17–3p* induced by Irx3 silences Olig2 in the p2 domain, forming the normal p2, pMN, and p3 progenitor domains. After MN generation, *miR-218* acts as a downstream effector of the Isl1-Lhx3 complex to establish and maintain MN identity. Among MN subtypes, *miR-9* seems to be involved in regulating MMC/LMC balance, whereas *miR-17∼92* directly targets components of E3 ubiquitin ligases to impact subcellular localization of PTEN through monoubiquitination in the LMC-MNs. (**B and C**) *Hoxa5* transcription in progenitor cells fluctuates, and translation of fluctuating transcripts at this time propagates noise, leading to strong stochastic variability. Two critical coherent forward loops involving *miR-27* are capable of preventing precocious Hoxa5 protein expression, thereby maintaining the critically sharp boundary between Hoxa5 and Hoxc8 protein expression in embryonic spinal cords. *Dicer* ^nerualΔ^: *Sox1Cre^+/-^; Dicer ^floxed^*, pMN: motor neuron progenitors, VZ: ventricular zone, IZ: intermediate zone, MZ: mantle zone. (**D**) MN-miRNA working models: *miR-218* is expressed in mature MNs to inhibit alternative neighboring genes. The potential *miR-218*-Slit-Robo autoregulatory loop is depicted. Subsequently, numbers of LMC motor neurons are reduced whereas MMC neurons are spared in *Dicer ^MNΔ^* and *miR-17 ~92 ^MNΔ^* spinal cords. Whereas surviving caudal brachial LMC motor neurons in *Dicer ^MNΔ^* mutants correctly express Hoxc6 and Hoxc8 factors, expression of motor pool markers (i.e. Pea3, Scip, and Nkx6.1) is significantly eroded, resulting in near absence of defined motor pool subtype identity. The miRNA candidates needed to maintain motor pool identity have yet to be identified. (**E**) Illustration of how *miR-17∼92* controls PTEN subcellular localization in developing LMC-MNs. This miRNA-mediated regulation modulates both target expression and target subcellular localization, providing LMC-MNs with an intricate defensive mechanism that controls their survival.

The patterning of the developing CNS relies on precise control of spatial and temporal expression of transcriptional regulators that progressively restrict cell potential to define ultimate neuronal identity and connectivity. In particular, establishment of sharp spatial boundaries and temporal transitions in gene expression is critical for correct neural patterning (Figures 2A and 3A). Recently, it has been suggested that small non-coding RNAs termed microRNAs (miRNAs), which are generated by cytoplasmic RNaseIII Dicer, might play an important role in sharpening and consolidating these transitions through their ability to inhibit mRNA translation (Bartel, 2018; Wolter et al., 2017). It has been proposed that more than one-third of the genes in the human genome might be regulated by miRNAs (Lewis et al., 2005). Interestingly, only a few examples of miRNA involvement in embryonic patterning and development have been reported thus far (Rajman and Schratt, 2017). This scenario might be due to two constraints: 1) many miRNAs belong to large families, with individual members functioning redundantly; and 2) since miRNAs might function primarily to sharpen developmental transitions, their deficiency may not lead to overt deficits or phenotypic transformation but instead to more subtle defects affecting subsets of cells found at developmental boundaries.

The developing spinal cord is an ideal system to uncover such subtle defects, as both the spatial and temporal boundaries that control cell specification in the ventral spinal cord, including for its spinal MNs, are well characterized (Figure 2). Therefore, miRNAs have been shown to play critical roles in the spinal cord, from neuronal progenitor patterning to cell fate specification and survival (Amin et al., 2015; Chen et al., 2011; Chen and Wichterle, 2012; Haramati et al., 2010; Hoye et al., 2017; Li et al., 2017; Thiebes et al., 2015; Tung et al., 2015). During DV patterning in the ventral spinal cord, the bistable switches of transcriptional cross-repressive genetic loops are particularly critical for resolving cell identity at progenitor domain boundaries (Balaskas et al., 2012; Jukam and Desplan, 2010). Interestingly, lineage tracing experiments in which *Cre* recombinase was knocked into the domain-specific patterning genes *Olig1*, *Olig2* or *Dbx1* (Chen et al., 2011; Dessaud et al., 2010; Dessaud et al., 2007; Wu et al., 2006) revealed that each of these factors is transiently expressed in a broad ventral spinal region spanning three or more neighboring progenitor domains. Thus, somewhat different to the transitional view of the static cross-repressive loop mentioned above (Figure 2A), the state of the cross-repressive genetic loops has to be malleable at the early developmental stage, and the initial broad expression of domain-specific determinants is then refined during development (Figure 3A). Repression of Olig2 in non-motor neuron progenitors appears to be partly achieved by temporal adaptation of spinal cells to Sonic hedgehog (Shh) signal. Clearance of Olig2 from the p3 domain (the most ventral neuronal progenitor domain) depends on induction of the repressor Nkx2.2 in response to sustained Shh signaling, whereas more passive loss of Olig2 expression in the p3 domain is proposed to be due to developmental de-sensitization of progenitors to the Shh signal (Dessaud et al., 2010; Dessaud et al., 2007; Wu et al., 2006). In contrast, the p2 domain (the third neuronal progenitor domain from dorsal to ventral) utilizes a TF-miRNA loop to consolidate its plastic state. By disrupting miRNA biogenesis during simulated DV patterning of differentiating ES cells both in cellulo and in vivo, it was revealed that *miR-17–3p*, a member of the *miR-17 ~92* cluster, is required to silence transient Olig2 expression in p2 progenitors (Chen et al., 2011) (Figure 3A). Thus, miRNA-mediated regulation of transcriptional programs might play a more general role in the refinement and positioning of spatial boundaries in developing neural tissue.

In addition to DV patterning in the neural tube, miRNAs also regulate the expression of TFs to fine-tune RC pattering of the spinal cord. Initial RC patterning of the neural tube leads to differential expression of *Hox* genes that contribute to the specification of MN subtype identity. *Hox* genes are clustered in genome loci that also contain miRNAs, including *miR-10* and *miR-196*. Intriguingly, *Hox* cluster-embedded miRNAs preferentially target *Hox* mRNAs (Yekta et al., 2008). Moreover, the *Hox-*cluster miRNAs are predominantly targeted to 3′ *Hox* (*Hox* 1 ~ 6) genes to ensure ‘posterior prevalence’ (Yekta et al., 2008). In the developing spinal cord of chick embryos, specific timing of *miR-196* expression is required for proper MN differentiation, which is mediated by downregulation of *Hoxb8*. Failure to clear Hoxb8 in a spatial and temporal manner abolishes MN generation (Asli and Kessel, 2010). This result highlights that both the spatial distribution and timing of Hox expression are pivotal for MN subtype diversification (Figure 3B). Coincidentally, although several *Hox* mRNAs, including *Hoxa5* and *Hoxc6*, are expressed in MN progenitors in a ‘noisy’ manner, the respective Hox proteins are not expressed in these progenitors and only become detectable in postmitotic MNs (Dasen et al., 2005; Li et al., 2017). In conditional neural *Dicer* mutants (*Sox1-Cre; Dicer^floxed^*, *Dicer ^NeuralΔ ^*) in which all mature miRNA production is abrogated in the CNS, Hoxa5 protein is precociously expressed at progenitors and the Hox5/8 boundary is expanded caudally, both in cellulo and in vivo (Figure 3B) (Li et al., 2017). Using *in silico* simulation, two feed-forward Hox-miRNA loops have been uncovered that account for the precocious and noisy Hoxa5 expression, as well as the ill-defined boundary phenotype of *Dicer* mutants. In this scenario, *miR-27* appears to be a major regulator coordinating the temporal delay and spatial boundary of Hox protein expression. This Hox-miRNA circuit filters transcription noise and controls the timing of protein expression to confer robust individual MN identity (Figure 3C). Thus, a miRNA-mediated mechanism regulating DV and RC patterning of the spinal cord provides two additional functional tuning mechanisms for establishing cell fate in MNs: 1) the coherent TF-miRNA feed-forward loop defines plastic cell fate at the early patterning stage to allow precise cell fate determination later (i.e., the Olig2/Irx3/*miR-17–3p* and Hoxc8/Hoxa5/*miR-27* loops); and 2) the retinoic acid-miRNA feed-forward loop is important for inhibiting fluctuations in Hox miRNA expression in the progenitors, as precocious protein expression at this stage may amplify stochastic effects, leading to noisy protein levels. This combined model represents a powerful strategy for achieving the precision and robustness of morphogen-mediated pattern formation (Figure 3C).

In addition to the role of miRNAs during neural progenitor patterning, miRNAs also play critical functions in postmitotic MNs. In the developing spinal cord, expression of *miR-218* is directly upregulated by the Isl1-Lhx3 complex to drive MN fate (Figure 3D). Loss of *miR-218* function leads to impaired generation of generic MNs, suggesting that this miRNA plays a crucial role in MN differentiation (Amin et al., 2015; Thiebes et al., 2015). Furthermore, mutant mice lacking *miR-218* die neonatally and exhibit neuromuscular junction defects, MN hyperexcitability, and progressive MN degeneration, recapitulating traits of motor neuron disease (Amin et al., 2015). These two studies highlight the predominant role of *miR-218* in mature MNs. However, *miR-218* does not seem to exert its functions by targeting a few specific genes. Instead, it appears to repress an array of transcripts in the alternative neighboring interneuron genes to maintain postmitotic MN cell fate and robust neuromuscular functions (Amin et al., 2015; Thiebes et al., 2015) (Figure 3D). Interestingly, *miR-218* is encoded by an intron of the extracellular matrix *Slit2* and *Slit3* genes and it inhibits expression of neural cell adhesion molecules Robo1 and Robo2 and multiple components of the heparan sulfate biosynthetic pathway responsible for normal vascularization of the retina (Small et al., 2010). Given that recent evidence indicates that the function of the Slit-Robo regulatory axis is also important for proper positioning of MNs and their exit points (Kim et al., 2019), it would be interesting to investigate if the *miR-218*-Slit-Robo autoregulatory loop is also important for MN axon pathfinding and targeting (Figure 3D).

Do miRNAs also play a role in postmitotic MN subtype diversification? Postmitotic MNs are diversified along the RC axis of the spinal cord to innervate corresponding target muscles. This process primarily relies on a regulatory network of Hox-TFs that translate neuronal identity into patterns of connectivity. Foxp1 is a crucial determinant of MN diversification and connectivity, interpreting the Hox regulatory network to control the formation of a topographic neural map. Although Hox/Foxp1 TFs have been recognized as principal regulators of subtype specification, a role for posttranscriptional regulation is also recognized. For instance, *miR-9* is transiently expressed in Foxp1^on^ LMC regions. Overexpression or knockdown of *miR-9* alters MN subtypes, switches columnar identities, and changes axonal innervations in developing chick spinal cords. *miR-9* modifies spinal columnar organization by specifically regulating Foxp1 protein levels, which in turn determine distinct MN subtypes (Otaegi et al., 2011) (Figure 3A). Additionally, deletion of the enzyme Dicer from all MNs using Olig2-Cre (*Dicer ^MNΔ ^*) revealed a preferential loss of many limb- and sympathetic ganglia-innervating spinal MNs (Chen and Wichterle, 2012). Furthermore, this disruption also led to defects in motor pool identity specification, yet the miRNA candidates to maintain motor pool identity have yet to be unveiled (Figure 3D). These results indicate that miRNAs are an integral part of the genetic program controlling MN survival and acquisition of subtype-specific properties. Subsequent study uncovered that the *miR-17 ~92* cluster is highly enriched in LMC-MNs. Furthermore, conditional MN *miR-17 ~92* deletion (*mi-17 ~92 ^MNΔ ^*) resulted in selective cell death of LMC-MNs. Mechanistically, *mir-17 ~92* not only suppresses PTEN expression, but also independently attenuates accumulation of nuclear PTEN, with this latter being a more potent apoptosis stimulator (Tung et al., 2015) (Figure 3E). Tellingly, reduced *miR-17 ~92* is accompanied by elevated nuclear PTEN in the spinal MNs of presymptomatic *SOD1^G93A^* mice, that is the most common mouse strain used as an ALS disease model. Selective dysregulation of the *miR-17 ~92*/nuclear PTEN axis in degenerating *SOD1^G93A^* LMC-MNs has been demonstrated in a double-transgenic embryonic stem cell system and, moreover, was recapitulated in human *SOD1^+/L144F^* induced pluripotent stem cell (iPSC)-derived MNs. Furthermore, overexpression of *miR-17 ~92* significantly rescues human *SOD1^+/L144F^* MNs, and intrathecal delivery of scAAV9 [self-complementary adeno-associated viral serotype 9]-*miR-17 ~ 92* improves motor deficits and survival in *SOD1^G93A^* mice (Tung et al., 2019). Therefore, *miR-17 ~92* either controls LMC-MN survival during embryonic development or confers MN subtype differential resistance to ALS-associated degeneration. Thus, similar to TFs, miRNAs contribute to neuronal progenitor patterning, cell fate specification, and long-term survival during MN differentiation (Table 1 and Figure 3).

**Table 1.** Proposed functions of microRNAs during spinal motor neuron (sMN) development

miRNA	Proposed roles in sMNs	Targets/interaction	Organism/cell models	Methods of genetic analyses	References
*miR-9*	*miR-9* promotes the switch from early-born to late-born motor neuron populations as well as MMC/LMC subtypes	*Foxp1*	Chicken	transient decoy of *miR-9* and overexpression of *miR-9* in ovo	(Luxenhofer et al., 2014; Otaegi et al., 2011)
*miR-17 ~ 92*	1. *miR-17–3p* carves p2/pMN boundary at progenitor stage 2. *miR-17 ~ 92* governs LMC-MN survival during development and degeneration in ALS	*Olig2* *PTEN/Ndfip1/Nedd4-2*	Mouse Mouse/human induced pluripotent stem cells	*CAGGS:Cre^ER^; Dicer ^floxed^* and *miR-17 ~ 92^-/-^* embryos *Olig2:Cre; miR-17 ~ 92 ^floxed^* embryos and *miR-17 ~ 92* overexpression in human ALS-iPSC derived MNs	(Chen et al., 2011) (Tung et al., 2015; Tung et al., 2019)
*miR-27*	*miR-27* filters Hox temporal transcription noise to confer boundary formation in the spinal cord	*Hoxa5*	Mouse and chicken	*Sox1:Cre; Dicer ^floxed^*, transient decoy of *miR-27* in ovo, and *mir-23 ~ 27 ~ 24^-/-^* embryos	(Li et al., 2017)
*miR-196*	*miR-196* confines the rostrocaudal axis in the neural tube	*Hoxb8*	Chicken	Transient decoy of *miR-196* in ovo	(Asli and Kessel, 2010)
*miR-218*	*miR-218* defines a neuronal gene network that is selectively tuned down in MNs to prevent neuromuscular failure and neurodegeneration.	Progenitor genes (*Sox21*, *Tead1*, etc.) Neighboring genes (*Slc6a1*, *Bcl11a*, *Foxp2*, etc.)	Mouse and chicken	Transient decoy of *miR-218* in ovo and *miR-218^-/-^* embryos	(Amin et al., 2015; Thiebes et al., 2015)

## MicroRNAs during motor neuron degeneration in SMA

Given by the versatile roles of miRNAs in regulating MN differentiation mentioned above, it is not surprising that miRNA dysregulation has been increasingly linked to MN-associated degeneration diseases, such as ALS and SMA. ALS-linked miRNAs have been intensively studied and elegantly reviewed elsewhere (Eitan and Hornstein, 2016; Emde et al., 2015). However, the role of miRNAs in SMA is just beginning to emerge. A detailed list of miRNAs implicated in SMA has been reviewed recently (Magri et al., 2018), which we summarize in Table 2. Here, we focus our discussion on some of the most recent studies linking specific miRNAs (i.e., *miR-2*, *miR-146a*, and *miR-23a*) to SMA pathogenesis and the potential of miRNAs as biomarkers and therapeutic targets.

**Table 2.** Proposed spinal muscular atrophy (SMA)-microRNA relationships

miRNA	Role in MN disease	Targets	Organism/cell models and expression profiles	Findings and proposed mechanism of SMA pathogenesis	References
*miR-2*	Neuronal development and function; correct NMJ functioning	CHRM2, m2R	*C. elegans* model, SMA mouse model. Decreased	Alters NMJ function	(O'Hern et al., 2017)
*miR-9 *^(1)^	MN dendritic outgrowth and synaptic function	Neurofilament heavy subunit (NEFH), REST, Map1b, MCPIP1	Mouse, patient fibroblasts, patient serum. Decreased in spinal cord, but increased in skeletal muscle	Dysregulated expression in MNs differentiated from ESCs. Regulation of MN subtype determination (FOXP1). miR-9 can delay neurite outgrowth in vitro and impair radial neuronal migration in embryonic mouse neocortex in vivo.	(Catapano et al., 2016; Haramati et al., 2010)
*miR-23a*	Neuroprotective properties; regulate axonal development; suppress skeletal muscle atrophy	Atrogin1, MuRF1 (maybe, no direct target experiment was verified by luciferase assay)	Patient induced pluripotent stem cells, SMA mouse model. Decreased	*miR-23a* can prevent the astrocyte-conditioned media-induced MN loss in vitro. In mice model, enhanced *miR-23a* expression via virus vector increased MN soma size and muscle fiber area, and reduced NMJ defects	(Kaifer et al., 2019)
*miR-100–5p*	Abnormal proliferation of neural progenitors; aberrant cell cycle	Potentially insulin-like growth factor one receptor (IGF1R)	SMN∆7 mouse neural stem cells from spinal cord. Decreased	Decreased *miR-100–5p* in SMAΔ7 mice neural stem cells induces high IGF1R, excessive proliferation of neural progenitors, and prevents appropriate exit of the cell cycle.	(Luchetti et al., 2015)
*miR-132* (possible)	Neuron dendritic outgrowth and synaptic plasticity; neovascularization, may cause ischemic pathology in both skeletal muscle and spinal cord of SMA model	Dysregulated expression due to TDP43 interaction with Dicer (amyotrophic lateral sclerosis data), p250GAP	SMA mice, patient serum. Decreased in spinal cord, but increased in skeletal muscle	Expression is dysregulated in TDP-43-deficient amyotrophic lateral sclerosis (ALS). Neuronal morphology and cognition in ALS. *miR-132* can delay neurite outgrowth in vitro and impair radial neuronal migration in embryonic mouse neocortex in vivo. Involved in synaptic plasticity. Process of neovascularization. Recent reports have highlighted vascular defects in both skeletal muscle and spinal cord of SMA patients.	(Catapano et al., 2016)
*miR-146*	MN loss caused by astrocyte-mediated pathology through NFκB signaling	GDNF, NOTCH2, GATA transcription factors	Patient induced pluripotent stem cells. Increased	*miR-146* levels are influenced both directly and indirectly by SMN1 levels. SMN re-expression decreases *miR-146a* levels nearly to control levels. The NFκB pathway is an inducer of *miR-146a*.	(Sison et al., 2017)
*miR-183*	Protein synthesis; axonal outgrowth	mTOR pathway	Mouse, cortical neurons, patient fibroblasts. Increased	Increased *miR-183* and reduced local axonal translation of mTOR in SMN-deficient neurons.	(Kye et al., 2014)
*miR-206 *^(2)^ (muscle-specific)	Myofiber formation; satellite cell differentiation; neuroprotective role in re-innervation of muscle endplates after acute nerve injury	Axis of HDAC4-FGFBP1, Pola1, BDNF	SMA mouse, patient serum. Increased in both spinal cord and skeletal muscle	Endogenously increased *miR-206*, with HDAC4 protein reduction and increased *FGFBP1* mRNA, activates neuroprotective mechanism in muscle cells to increase re-innervation of muscle endplates.	(Catapano et al., 2016; Valsecchi et al., 2015)
*miR-335–5p*	Control of differentiation or self-renewal of mouse ESCs	MEST, OCT4, RB1	SMN∆7 mouse neural stem cells, human induced pluripotent stem cells. Decreased	Possible epigenetic regulation through methylation to affect cell differentiation.	(Luchetti et al., 2015; Murdocca et al., 2016)
*miR-375*	Neurogenesis and protects neurons from apoptosis in response to DNA damage.	P53, PAX6, CCND2	Human neural progenitor cell cultures. Decreased	MNs from an SMA patient have shown reduced levels of *miR-375*, elevated p53 protein levels, and higher susceptibility to DNA damage-induced apoptosis.	(Bhinge et al., 2016)
*miR-431*	Regulation of motor neuron axon neurite outgrowth	Chondrolectin (Chodl): a type one transmembrane protein and member of the c-type lectin domain-containing family	Mouse MN culture, patient fibroblasts/induced pluripotent stem cells. Increased	Increased *miR-431* regulates motor neuron neurite length by targeting chondrolectin involved in motor neuron axon outgrowth.	(Wertz et al., 2016)

^1 ^*miR-9*/9* is another microRNA potentially implicated in motor neuron disease. It has been linked to the loss of spinal motor neurons that leads to SMA. ^2 ^Since *miR-206* is required for efficient regeneration of neuromuscular synapses after acute nerve injury, this scenario probably accounts for its salutary effects in SMA.

SMA is an autosomal recessive neurodegenerative disease characterized by devastating muscular atrophy attributable to progressive spinal MN degeneration. Although SMA is a relatively rare disease, with an estimated worldwide incidence of one in 6,000 ~ 10,000 newborns, it is notable that SMA is the second most common autosomal recessive disease after cystic fibrosis and is the most common monogenic defect leading to infant mortality (Lunn and Wang, 2008). SMA is caused by reduced levels of the 38 kDa Survival Motor Neuron (SMN) protein due to deletion or mutation of the *Survival of Motor Neuron 1* (*SMN1*) gene. In humans, SMN protein is actually encoded by two genes: *SMN1* and highly homologous *SMN2*, which essentially differs by one nucleotide (C→T) in exon 7. This critical difference results in preferential exclusion of exon seven from most *SMN2* transcripts, termed SMN△7. As a consequence, *SMN2* generates ~10% of full-length (FL) SMN mRNAs and their product-functional SMN proteins (Burghes and Beattie, 2009). As these FL-*SMN2* transcripts can partially compensate for loss of *SMN1*, it is reasoned that the FL-*SMN2* transcript copy number may determine phenotypic severity in SMA patients. Therefore, SMA is caused by loss of the *SMN1* gene and disease manifestation is partially reflected by the degree of *SMN2* gene compensation (Burghes and Beattie, 2009). However, recent studies have indicated that additional modifiers might also be involved in modulating SMA clinical severity (Crawford et al., 2012).

SMN is ubiquitously expressed in almost all kinds of somatic cells, with a distribution in both the cytoplasm and nucleus (Burghes and Beattie, 2009). Notably, the most appreciated canonical role of SMN is to serve as an essential ribonucleoprotein (RNP) for mRNA splicing. SMN protein is embedded in a complex with seven Gemins and UNR-interacting protein (UNRIP) that loads Sm protein onto nascent uridine-rich noncoding RNAs (U-snRNAs) upon their export to the cytoplasm, thereby creating small nuclear RNPs (snRNPs) that form spliceosomes in the nucleus (Burghes and Beattie, 2009; Dostie et al., 2003). In addition to facilitating snRNP assembly, the SMN complex has a role in assisting arginine methylation of some splicing-related proteins that are involved in pre-mRNA splicing (Meister and Fischer, 2002; Pellizzoni et al., 2002). Studies on SMA animal models have also revealed a direct correlation between the ability to assemble snRNPs and SMA phenotypes, and delivery of mature snRNPs even without the SMN component is sufficient to rescue SMA phenotypes (Gabanella et al., 2007; Winkler et al., 2005; Workman et al., 2009). This outcome implies that SMN protein levels might affect splicing of SMN pre-mRNA to include exon seven through an autoregulatory loop, thereby influencing a general process of snRNP biogenesis (Jodelka et al., 2010). In addition to the canonical role of SMN protein in the splicing machinery, some studies have also highlighted the multilayer functions of SMN. For example, assembly of SMN protein is also involved in a number of essential cellular pathways, including DNA repair and protein/mRNA transportation along MN axons (Donlin-Asp et al., 2016; Fallini et al., 2012; Murray et al., 2015; Tu et al., 2017). Collectively, studies to date support that loss of SMN-RNP complex assembly and its activity results in a series of different cellular pathways that lead to SMA. However, it is still unclear if SMA pathology is due to a particularly vulnerable pathway or a combination of dysregulated effects. In addition, it is still unclear how a deficiency in ubiquitously expressed SMN can result in selective MN degeneration.

Deletion of the miRNA biogenesis enzyme Dicer in MNs either using Olig2-Cre or ChAT-Cre results in MN degeneration that mimics certain hallmarks of SMA and ALS (Chen and Wichterle, 2012; Haramati et al., 2010; Tung et al., 2019). Large-scale miRNA profiling platforms have identified miRNAs that are significantly dysregulated in MN disease. Dysregulated MN-specific miRNAs are potential culprits in spinal MN mortality. In this scenario, some important developmentally-regulated (i.e. *miR-9 *and *miR-133*) and MN-enriched miRNAs (i.e. *miR-218* and *miR-17 ~92*) have been implicated as being important for SMA and/or ALS onset or progression (Amin et al., 2015; Hoye et al., 2017; Kye and Gonçalves, 2014; Tung et al., 2015; Tung et al., 2019). Furthermore, other neuronal-enriched miRNAs also seem to be involved in spinal diseases, such as *miR-21*/*miR-431*/*miR-138* for axonal regeneration of sensory neurons or *miR-196a* in spinal and bulbar muscular atrophy (SBMA) (Liu et al., 2013; Miyazaki et al., 2012; Strickland et al., 2011; Wu and Murashov, 2013). These findings raise the possibility that miRNAs might serve as: 1) important regulators of SMN-mediated pathways; and 2) potential markers reflecting SMA pathology.

**Figure 4.**
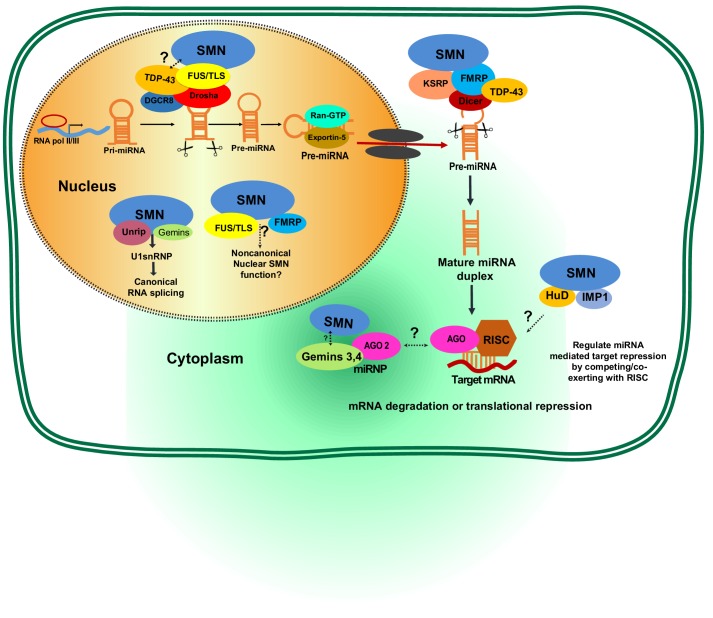
Potential involvement of SMN RNA-binding proteins (RBPs) in regulating microRNA biogenesis and function. During the first step of miRNA biogenesis in the nucleus, pri-miRNA is processed by a microprocessor complex comprising Drosha and DiGeorge syndrome chromosomal region 8 (DGCR8) to produce hairpin-shaped pre-miRNA. Pre-miRNA is then exported to the cytoplasm by exportin-5 and further cleaved by Dicer. During nuclear and cytoplasmic processing of primary and precursor levels of miRNA, several RBPs—including TAR DNA-Binding Protein 43 (TDP-43), fused sarcoma/translocated in liposarcoma (FUS/TLS), KH-type splicing regulatory protein (KSRP), and Fragile X mental retardation protein (FMRP)— are known to link the SMN complex to the Drosha/DGRC8 complex and/or Dicer. Subsequently, the miRNA duplex is unwound into a single strand that is then loaded into argonaute proteins (AGO) to form the RNA-induced silencing complex (RISC). The miRNA-RISC complex plays a crucial role in post-transcriptional repression of target mRNA expression. Notably, during maturation and action of functional miRNAs either in the cytoplasm or nucleus, the SMN complex is known to interact with several RBPs including FMRP, HuD, and insulin-like growth factor 2 mRNA-binding protein 1 (IMP1). However, it remains to be established if SMN-RBP is involved in miRNA-mediated silencing.

Despite this evidence of a role for miRNAs in SMA disease progression, it remains unclear if dysregulated miRNAs are the direct pathogenic cause or a consequence of SMA progression. Given that SMN protein per se does not contain known RNA- or miRNA-binding domains (Bertrandy et al., 1999; Ravanidis et al., 2018), the SMN protein complex might instead interact with the miRNA biogenesis pathway to regulate miRNA production (Ravanidis et al., 2018). For example, Gemin3 and Gemin4 of the SMN complex can assemble with a number of miRNAs to form a miRNA-binding RNP (miRNP) (Dostie et al., 2003; Mourelatos et al., 2002) (Figure 4). This miRNP complex can further bind to Argonaute 2 (AGO2) that functions as a core protein in the RNA-induced silencing complex (RISC) mediating miRNA biogenesis as well as mRNA posttranscriptional regulation (Cauchi, 2010). Yet SMN does not seem to bind to miRNP directly, if the miRNP is functionally important in SMA pathology still needs to be further investigated. Interestingly, in addition to the role of SMN in RNA processing, recent studies have uncovered unexpected interactions of SMN with miRNA biogenesis proteins including fragile X mental retardation protein (FMRP), KH‐type splicing regulatory protein (KSRP) and fused in sarcoma/translocated in liposarcoma (FUS/TLS) (Piazzon et al., 2008; Sun et al., 2015; Tadesse et al., 2008; Yamazaki et al., 2012) (Figure 4). Thus, SMN is engaged in miRNA–RBP complexes and likely regulates miRNA biogenesis and metabolism. Deficiency of SMN may alter MN-specific miRNAs or miRNPs, resulting in MN death (Cauchi, 2010; Nelson et al., 2004). In Table 3, we list SMN-associated RBPs and their roles in miRNA biogenesis and functions. In Figure 4, we illustrate potential mechanisms by which SMN interacts with various RBPs to regulate miRNA biogenesis and function. In summary, the proposed mechanisms by which SMN-associated RBPs might be involved in miRNA biogenesis include: (1) facilitating recruitment of Drosha to specific miRNAs; (2) binding to components of the Drosha and Dicer complexes; (3) acting as regulators to RISC complex (Fallini et al., 2014; Gonçalves et al., 2018; Ravanidis et al., 2018). Consequently, alteration of the SMN-RBP complex caused by SMN deficiency in the SMA pathological background might lead to dysregulated processing of miRNAs and pre-mRNA splicing. Identification of SMN-associated RBPs involved in transport and processing of different miRNAs could help explain SMA pathogenesis and reveal novel therapeutic targets.

**Table 3.** Proposed SMN-associated RNA-binding proteins involved in microRNA pathways.

SMN-associated RBPs	RBP interaction assay	General functions in posttranscriptional regulation of mRNA	Proposed roles in miRNA metabolism/processing	Neuronal modeling mechanism	References
TAR DNA-binding protein-43 (TDP-43)^a,b^	FLAG-Drosha or FLAG-TDP-43 interacts in nuclear extracts from HEK293T cells. Partial colocalization of TDP-43 and SMN in MNs.	Transcriptional regulation, pre-mRNA splicing, mRNA transport and translation	1. Integrating with the Dicer and Drosha complexes 2. Regulating pre-miRNA biogenesis	Neuron differentiation, neuronal plasticity, synapse formation, neurite outgrowth	(Kawahara and Mieda-Sato, 2012; Wang et al., 2002)
Fused sarcoma/translocated in liposarcoma (FUS/TLS)^a,c^	IP and GST pulldown SMN in HeLa nuclear extracts and mouse neuroblastoma (N2a) using FUS antibody.	Transcriptional regulation, pre-mRNA splicing, mRNA transport and translation	Recruiting the Drosha complex	Synapse formation, neuronal plasticity, neurite outgrowth, neuronal differentiation and proliferation	(Morlando et al., 2012; Sun et al., 2015; Yamazaki et al., 2012)
Fragile X mental retardation protein (FMRP)	IP and GST pulldown SMN complex in human neuroblastoma SH-SY5Y cells using FLAG-tagged FMRP antibody. IP antibodies specific to human FMRP were used to pull down Dicer and associated miRNAs in an EBV-transformed human B cell line. IP was performed from total HeLa cell lysates with a monoclonal antibody to eIF2C2.	mRNA stability, translation inhibition	Integrating with AGO and Dicer in RNA-induced silencing complex (RISC)^d^	Synapse formation, neuronal plasticity, neurite outgrowth, neuronal differentiation and proliferation	(Edbauer et al., 2010; Jin et al., 2004; Piazzon et al., 2008)
KH-type splicing regulatory protein (KSRP)	Endogenous KSRP was IP from N2a cells with SMN Tudor domain. KSRP and SMN colocalize in neuronal processes foci	mRNA stability, translation inhibition	Interacting with Drosha and Dicer complexes to regulate miRNA maturation and mRNA decay	KSRP is expressed in the nervous system in both neurons and glia and plays a role in control of neuronal mRNA stability and rate of axonal outgrowth	(Amirouche et al., 2014; Tadesse et al., 2008; Trabucchi et al., 2009)
HuD (ELAV-like protein 4)	SMN and the neuron-specific RBP HuD interact biochemically in cultured primary cortical neurons, MN1-cells, cultured mouse MNs, rat brain extracts. Genetic evidence from zebrafish embryos support that the interaction between SMN and HuD is critical for MN development.	mRNA stability, transport, translation, mTOR pathway	Interacting with RISC^d^ to regulate miRNA function	Axon development, maintenance, and plasticity	(Fukao et al., 2014; Hao le et al., 2017; Loffreda et al., 2015; Sosanya et al., 2013)
IMP1 (IGF2BP1; ZBP1), insulin-like growth factor 2 mRNA-binding protein 1	IP and GST pulldown SMN in brain lysates extracts using IMP1 antibody.	Promotes mRNA stability by preventing miRNA-mediated silencing	Interacting with AGO2 to regulate miRNA function	Axon development	(Fallini et al., 2014; Gardiner et al., 2015) (Degrauwe et al., 2016)

a. TDP-43 and FUS/TLS proteins bind to pre-mRNA molecules and determine their fates by regulating splicing, transport, stability and translation. b. The RNA-binding protein, TDP43, biochemically interacts with the miRNA processing enzyme Drosha, raising the possibility that TDP-43 may play a role in miRNA processing.

c. FUS/TLS promotes biogenesis of specific miRNAs, including *miR-132*, *miR-134*, and *miR-9*. d. Additional proteins associated with RISC include MOV10 and Hu-AntigenR.

A series of studies has shown dysregulation of specific miRNAs in SMA, and several of these studies have proposed potential links between aberrant miRNA expression and SMA pathophysiology in a diverse array of SMA animal models and patient samples, including neurons from nematodes, neurons and muscles from mice, as well as fibroblasts and serum from human patients (Table 2). Furthermore, transcriptome profiling has uncovered a number of miRNAs associated with MN survival, synapse formation, ER stress, and ribosomal RNA binding in models of SMA (Kye and Gonçalves, 2014; Vanderweyde et al., 2013; Viswambharan et al., 2017). It remains under debate if dysregulated miRNAs and the corresponding miRNA-mediated target responses are cell-context dependent (i.e., specifically in MNs or in all neurons of the spinal cord), given that SMN is expressed ubiquitously and compromised expression of SMN may selectively affect miRNA homeostasis in different tissues (Magri et al., 2018). In this regard, a very recent study aimed to identify miRNAs that are differentially regulated in SMA from iPSC-derived MNs (Kaifer et al., 2019). In that study, Kaifer et al. only identified a subset of 16 miRNAs whose expression is significantly reduced more than 2-fold in SMA MNs when compared to control MNs. However, using a scAAV9 viral vector to reintroduce *mir-23a* into the Smn^2B/-^ SMA mouse model increased MN size, reduced neuromuscular junction (NMJ) pathology, and extended survival. Although the detailed mechanisms underlying how *miR-23a*-mediated target pathways lead to SMA pathology have yet to be characterized, these findings suggest that only a cohort of miRNAs might cause the MN vulnerability in SMA and identification of those miRNA culprits and their targets could provide a new treatment strategy for SMA.

In addition to MNs, evidence for roles for miRNAs in muscle and glial cells that contribute to SMA is also emerging. By using both mouse and nematode models of SMA, O' Hern et al. showed that reduced SMN activity specifically affects *miR-2* expression, which in turn causes MNs to produce more m2R, a receptor for acetylcholine (O'Hern et al., 2017). This result also indicates that reduced SMN levels might lead to NMJ dysfunction in MNs and suggests that dysregulation of neurotransmission could be another critical causative factor for SMA pathology. Interestingly, it was recently shown that SMN protein levels in astrocytes affect both directly and indirectly altered expression levels of *miR-146a* (Sison et al., 2017). The proposed mechanism for that astrocyte-mediated SMA pathology was linked to increased levels of *miR-146a* in the SMNΔ7 mouse spinal cord. Although the precise targets of miR*-146a* have not yet been identified, treating iPSC-derived MNs with synthesized *miR-146a* molecules was sufficient to induce significant MN loss. This outcome indicates that SMA astrocyte-secreted *miR-146a* might lead to non-cell-autonomous loss of MNs. In the future, it is tantalizing to test gene therapy using miRNA cocktails from astrocytes and/or muscles cells, together with MN-intrinsic miRNAs to mitigate the SMA symptom.

## MicroRNAs as SMA biomarkers

Since SMA is a monogenetic disease, designing treatment strategies that restore SMN function in patients is a rational approach. Two undergoing SMA treatment trials either use antisense oligonucleotides or virus-mediated gene therapy exhibited promising outcomes (Finkel et al., 2017; Mendell et al., 2017). These therapeutic approaches both aim to increase SMN protein production in the spinal cord to restore motor function and survival. Mendell et al. used single intravenous administration of nonreplicating scAAV9 adenovirus vector carrying a human wild type SMN1 sequence. In contrast, Finkel et al. applied an antisense approach that was designed to enhance inclusion of exon seven in SMN2, which involved repetitive intrathecal administration of the antisense oligonucleotide drug Nusinersen. While there is cautious optimism regarding progress in developing SMA therapies, several new critical questions have arisen, particularly with regard to identifying measures of reliable outcomes in preclinical and clinical trials, given that: 1) intrathecal delivery is relatively invasive; 2) treatments remain prohibitively expensive; and 3) it remains unclear whether patients’ responsive or non-responsive reactions to treatments reflects SMA heterogeneity. Accordingly, researchers have been prompted to identify more authentic biomarkers of SMA to facilitate patient classification, to follow disease progression, and to better monitor responses to therapeutic approaches using minimally invasive procedures. Reliable biomarkers would not only help categorize heterogeneous clinical types of SMA patients into homogeneous prognostic groups, but would also improve statistical power of clinical trials, reduce trial durations and costs, and reveal therapeutic effects in specific types of SMA patients (De Paola et al., 2019; Viswambharan et al., 2017).

Currently, the efficacy of SMA treatments is largely measured by clinical outcomes, including motor function, electrophysiological tests, and dependency on mechanical ventilation (Finkel et al., 2017; Mendell et al., 2017; Mercuri et al., 2018). However, fluctuating inter-rater or intra-rater variabilities of motor function measurement is problematic and are usually confounded by diverse care regimens and age groups. It is conceivable that molecular biomarkers may represent more objective measures of treatment efficacy. Quantification of SMN mRNA or protein levels has served as the most commonly used molecular biomarker of SMA, but it does not necessarily correlate with disease severity and may not reliably reflect disease progression (Sumner et al., 2006; Wadman et al., 2016). MiRNAs have gained attention as an easily accessible biomarker due to their characteristic of being clinically detectable in many biofluids - such as cerebrospinal fluid, serum/plasma, saliva, and urine - using noninvasive methods (Viswambharan et al., 2017). Indeed, miRNAs are now being used as novel clinical biomarkers for the prognosis of several diseases, including colorectal cancer, acute leukemia, coronary artery diseases, as well as neurodegenerative disorders (Xu, 2012). Furthermore, miRNAs have also been proposed as potential biomarkers in several clinical trials for neuromuscular disorders (Alexander and Kunkel, 2015; Toivonen et al., 2014). Altered miRNA expression has been reported for spinal cord, brain, iPSCs, muscle, blood and cerebrospinal fluid of ALS patients (Hawley et al., 2017; Tung et al., 2019). As SMN is known to be involved in miRNA expression, circulating miRNAs may be used as diagnostic or prognostic biomarkers to reflect SMA pathology or therapeutic effects. As summarized in Table 2, *miR-9*, *miR-132*, *miR-206*, *miR-183* and *miR-375* have been proposed as potentially reliable SMA biomarkers (Israeli et al., 2016; Perry and Muntoni, 2016; Viswambharan et al., 2017). However, it remains to be tested if any of these markers could serve as appropriate indicators of the efficacy of the aforementioned antisense oligonucleotide- or scAAV9-mediated treatments. Answering this question is imperative, as early identification of good patient responders by means of multiple sensitive outcome measures can decrease the cost of innovative therapeutics (Kolb et al., 2016; Taga and Maragakis, 2018). Notably, a recent phase 3 study of an antisense oligonucleotide treatment for severe infantile SMA revealed quite distinct responses among patients (Finkel et al., 2017). In that study, the only factor identified as influencing treatment response was the time between recording the first symptoms in type I SMA patients and initial delivery of the antisense oligonucleotide. However, this factor would not seem to satisfactorily explain the wide variation in patient outcomes. Identification of a set of reliable miRNA markers from serum/cerebrospinal fluid that can be correlated with the Hammersmith Functional Motor Scale, a commonly used clinical parameter in SMA trials, might help stratify the variability among patients (Catapano et al., 2016; Gidaro and Servais, 2019).

## Conclusion and future perspective

The relevance of miRNAs to development and disease contexts has been gaining increasing attention over the past two decades. Many miRNA knockout mouse lines have been shown to manifest significant neural development phenotypes (Bartel, 2018), and miRNAs were shown to be sufficient to facilitate reprogramming of fibroblasts into neurons, including into spinal MNs (Abernathy et al., 2017). In this review, we summarize the reported functions of miRNAs in the development and degeneration of spinal MNs. Although ~5 miRNAs are highly or specifically expressed in MNs, only two mouse knockout lines (i.e., *miR-17 ~92^MNΔ^* and a *miR-218* double knockout) have been characterized in detail. Thus, it would be prudent to further verify the proposed roles of *miR-196* and *miR-9* in the regulation of Hoxb8 and Foxp1 by examining their respective knockout mouse lines (Shibata et al., 2011; Wong et al., 2015). It is relatively tedious to generate miRNA knockout mice as many miRNAs have evolved duplicated paralogs in mammals (Park et al., 2012), hindering functional studies of miRNAs in mouse models. Fortunately, this hurdle can now be circumvented by CRISPR-Cas9 technology. Accordingly, we envision that further mechanistic insights into the miRNA-TF network of cell fate specification and maintenance during spinal MN development will be revealed by mouse genetic approaches in the near future.

Despite miRNAs reportedly serving as potential biomarkers for cancer prognosis and progression (Hayes et al., 2014), their potential applications in neurodegenerative disease remain to be fully validated. In the case of SMA discussed here, emerging evidence indicates that SMN deficiency can impact non-neuronal organ systems, which may be reflected by altered levels of specific miRNAs in serum (Hamilton and Gillingwater, 2013; Hua et al., 2015). However, critical challenges remain, such as unraveling inconsistencies due to human subject variability and technical issues related to the relative fragility of miRNAs (Basak et al., 2016; Viswambharan et al., 2017). Although SMA-iPSCs might overcome some of these difficulties, it will be critical to recruit a large cohort of patients to validate the authenticity of miRNA biomarkers and further promote their utility.

Finally, twenty years after the breakthrough discovery illustrating how RNA interference can be used to silence certain genes, the U.S. Food and Drug Administration has approved the first drug utilizing this method for adult clinical treatment of a rare disease (i.e., hereditary transthyretin-mediated amyloidosis). However, as for antisense oligonucleotide treatment in SMA, the cost is prohibitive. Other significant challenges that must be overcome before routine utilization of miRNA-based therapy in neurodegenerative diseases include establishing the best route of delivery and ensuring miRNA therapeutics can cross the blood-brain barrier. In addition, given that miRNAs often act through multiple pathways, the risk of off-target effects must be addressed, especially since they are frequently cited as a side-effect of molecular therapies. Lastly, the timing of miRNA regulation according to cellular developmental status must also be taken into consideration, with miRNA levels being manipulated at a precise time-point with an optimal dosage.

In conclusion, miRNAs are just as critical as TFs for MN development, and miRNA dysregulation likely plays an important role in SMA pathogenesis and in determining the selective vulnerability of MNs. Considering the rapid pace of our expanding knowledge on non-coding RNAs and the introduction of cutting-edge single-cell RNA/ATAC sequencing technology (Delile et al., 2019), research into the role of miRNAs in neural development and degeneration has just begun.
